# The Effect of Waste Engine Oil and Waste Polyethylene on UV Aging Resistance of Asphalt

**DOI:** 10.3390/polym12030602

**Published:** 2020-03-06

**Authors:** Chao Peng, Chong Guo, Zhanping You, Fang Xu, Wenbo Ma, Lingyun You, Tianjun Li, Lizhen Zhou, Shifan Huang, Hongchao Ma, Li Lu

**Affiliations:** 1 Faculty of Engineering, China University of Geosciences, Wuhan 430074, China; guochong@cug.edu.cn (C.G.); xufang@cug.edu.cn (F.X.); liyou@mtu.edu (L.Y.); huangshifan@cug.edu.cn (S.H.); 1981393538@cug.edu.cn (H.M.); 1201910375@cug.edu.cn (L.L.); 2 Department of Civil and Environmental Engineering, Michigan Technological University, Houghton, MI 49931-1295, USA; zyou@mtu.edu; 3 Key Laboratory of Road Structure and Material of Ministry of Transport (Changsha), Changsha University of Science & Technology, Changsha 410004, China; mawenbo@xtu.edu.cn; 4 College of Civil Engineering and Mechanics, Xiangtan University, Xiangtan 411105, China; 5 Ministry of Education, Engineering Research Center of Rock-Soil Drilling & Excavation and Protection, China University of Geosciences (Wuhan), Wuhan 430074, China

**Keywords:** waste engine oil, waste polyethylene, ultraviolet aging, gel permeation chromatography, Fourier transform infrared spectroscopy, atomic force microscopy

## Abstract

Waste engine oil (WEO) and waste polyethylene (WPE) are two common wastes, which are easy to pollute the environment. As the primary material in road construction, natural asphalt is a non-renewable energy source and asphalt is vulnerable to ultraviolet (UV) radiation during the service life. It results in degradation of asphalt pavement performance. In this paper, 2 wt % to 8 wt % of WEO and WPE were used to modify asphalts and the UV aging simulation experiment was carried out. The physical parameters of asphalts before the UV aging experiment show that the asphalt containing 4 wt % WPE and 6 wt % WEO mixture (4 wt % WPE + 6 wt % WEO) has similar physical properties with that of the matrix asphalt. Besides, gel permeation chromatography (GPC) verifies that the molecular weight distribution of the asphalt containing 4 wt % WPE + 6 wt % WEO is close to that of the matrix asphalt. The storage stability test shows that 4 wt % WPE + 6 wt % WEO has good compatibility with the matrix asphalt. The functional groups and micro-morphology of asphalts before and after the UV aging experiment were investigated by Fourier transform infrared spectroscopy (FTIR) and atomic force microscopy (AFM). FTIR results display that 4 wt % WPE + 6 wt % WEO can effectively reduce the formation of carbonyl and sulfoxide functional groups. AFM shows that 4 wt % WPE + 6 wt % WEO can also retard the formation of a “bee-like” structure in asphalt after the UV aging experiment. Based on the above results, it can be concluded that WEO and WPE mixture can replace part of asphalt and improve the UV aging resistance of asphalt.

## 1. Introduction

Asphalt pavement has been widely used in road engineering construction due to its high strength, durability, and impermeability. As the primary material in road construction, asphalt is a non-renewable energy source [1]. At the same time, the demand for asphalt increases year by year with the rapid development of road construction. Therefore, it is urgent to find materials that can replace part of the asphalt [2]. Waste oil and waste plastic are two wastes in industrial production and daily life. Waste oil can cause harm to the environment and waste plastic becomes the primary source of “white pollution” because of hard degradation. To make things worse, the waste plastic releases a large amount of harmful gas during incineration treatment.

Recently, many pieces of research have been focused on using waste oil or waste plastic to modify asphalt [3,4]. They found that the main component of waste engine oil (WEO) is similar to the lightweight component of asphalt. According to the principle of “like dissolves like”, WEO can be used to modify asphalt [5]. Jia et al. [6] used WEO residues to partially replace asphalt. They found that the addition of WEO improves low-temperature performance. However, it also impaired the high-temperature resistance of the asphalt and its resilience at the same time. Liu et al. [7] used WEO to modify asphalt. They found that the addition of WEO is beneficial to prevent asphalt aging and improving fatigue performance, but WEO has a negative effect on the rutting resistance of asphalt. Villanueva et al. [8] prepared the modified asphalt with used lubricating oil (or engine oil). They found that used lubricating oil improves the anti-cracking performance at low-temperatures and reduces the quality loss of asphalt after rolling thin film oven test (RTFOT). Liu et al. [9] mixed WEO with the matrix asphalt and styrene–butadiene–styrene (SBS) modified asphalt, respectively. They revealed that the appropriate contents of WEO increase the fatigue resistance and temperature sensitivity of asphalt. In addition, WEO affects the matrix asphalt more significantly than SBS modified asphalt. Qurashi et al. [10] studied the aging performance of WEO as a rejuvenator when it is incorporated into asphalt. They discovered that 2–4 wt % of WEO eliminates the negative influence of aging on asphalt performance and improves the flexibility and elasticity of asphalt. DeDene et al. [11,12] added WEO into the reclaimed asphalt binder (RAB) and found that WEO can restore the performance of RAP. Meanwhile, WEO can also improve the low-temperature properties of asphalt mixtures blended with RAB.

Waste polyethylene (WPE), as another asphalt modifier, has also been investigated by many researchers. Polacco et al. [13] used several polyethylenes and polyethylene-based polymers to modify vacuum-distilled asphalt. They found that linear low-density polyethylene had good compatibility with asphalt and enhances the mechanical properties of asphalt. Attaelmanan et al. [14] used high-density polyethylene (HDPE) as an asphalt modifier. They discovered that HDPE and asphalt are compatible to a certain extent and HDPE improves the high-temperature shear resistance of asphalt. By adding HDPE, the moisture sensitivity and temperature sensitivity of asphalt can be reduced. Hu et al. [15] used WPE from packaging as a modifier of matrix asphalt and found that WPE modified asphalt has better deformation resistance than matrix asphalt. Fang et al. [16] blended WPE with the matrix asphalt. They found that WPE does not react with asphalt and WPE modified asphalt still had excellent high-temperature stability after the oxidation aging test. Priyansh et al. [17] incorporated the pulverized WPE into the asphalt by the dry mixing method and used a dynamic shear rheometer to evaluate the viscoelasticity of WPE modified asphalt. They found that WPE improves the adhesion of the asphalt in the short heating time during the mixing process. Fuentes-Auden et al. [18] used recycled polyethylene (RPE) to modify 150/200 penetration grade asphalt and discovered that RPE can improve rutting and the fatigue cracking resistance of asphalt binder.

Based on the above research related to WEO and WPE applications, it reveals that WEO is beneficial for the low-temperature performance of asphalt and WPE improves the high-temperature stability of asphalt. However, the high- or low-temperature properties of asphalt modified by the WEO and WPE mixture (WEO/WPE) are still unknown.

During the service life of asphalt pavement, asphalt surface aging is vulnerable to take place due to the ultraviolet (UV) radiation from the sunlight. UV can change the chemical composition and chemical structure of asphalt and weaken the performance of asphalt pavement [19,20,21,22]. Bocci et al. [23] designed a UV aging simulation experiment in the laboratory. They found that increasing UV radiation intensity aging time has a similar effect on asphalt with prolonging UV aging time. Peng et al. [24,25,26] studied the effect of layered double hydroxides (LDH) containing p-methyl cinnamic acid anions (Zn-Al-PMCA-LDH), LDH containing 4,4′-stilbene dicarboxylic acid anions (Zn/Al-SA-LDH), and LDH containing 2-hydroxy-4-n-octoxy-benzophenone anions (Zn/Al-HNOB--LDH) on asphalt UV aging resistance. The results show that Zn-Al-PMCA-LDH, Zn/Al-SA-LDH, and Zn/Al-HNOB--LDH have a synergistic effect on reflecting and absorbing UV light. Cao et al. [27] used nitric acid to etch layered double hydroxides (LDHs) and discovered that LDHs after etching treatment enhances the anti-UV aging properties of asphalt much more than LDHs without etching treatment. From the above studies, it can be seen that increasing UV aging resistance of asphalt is essential to prolong the service life of asphalt pavement. To our best knowledge, the UV aging resistance of asphalts modified by the WEO/WPE mixture has not been investigated.

In this work, the physical parameters of asphalts modified by WEO, WPE, and WEO/WPE mixture were tested. Then, the molecular weight distribution characteristics of asphalts were analyzed by gel permeation chromatography (GPC). The compatibility between the modifiers and the matrix asphalt was evaluated by the storage stability test. Subsequently, an ultraviolet aging simulation experiment of asphalts was carried out. The functional groups of asphalts before and after UV aging were analyzed by Fourier transform infrared spectroscopy (FTIR). Finally, the micro-morphology of asphalts before and after UV aging was observed by atomic force microscopy (AFM).

## 2. Experiments

### 2.1. Experimental Materials

**Figure 1:**
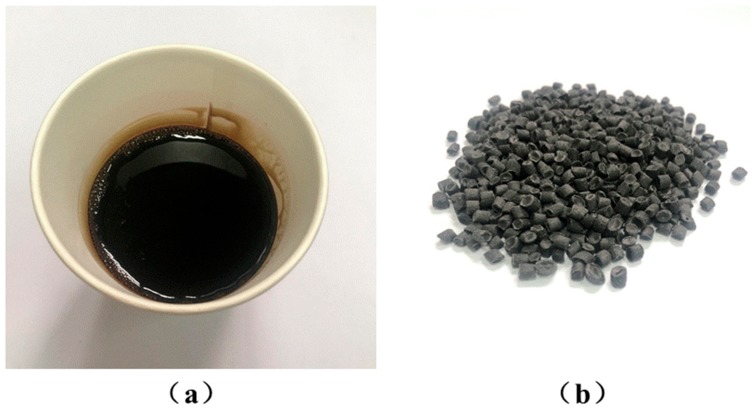
(**a**) Waste engine oil; (**b**) waste polyethylene particles.

The selected 90 penetration grade matrix asphalt was produced by Maoming Petrochemical Company of Guangdong Province, Maoming, China. A 5W-30 type of WEO was collected by Wuhan Junying Automobile Repair Factory in Hubei Province, China. It is a brown liquid, which is shown in Figure 1a. Waste polyethylene packaging plastic bags were treated by hot-melt extrusion and crushing process to prepare WPE particles, which are shown in Figure 1b.

### 2.2. Preparation of WEO and WPE Modified Asphalts

Firstly, the matrix asphalt was heated to 170 °C. A certain mass ratio of WEO and WPE was blended with the heated matrix asphalt manually for 15 min. Then, a high-speed shear mixer was used to stir the asphalt at the speed of 4000–5000 r/min for 30 min at 170 °C. The mass ratios of the matrix asphalt and different modifiers in prepared asphalt samples are shown in Table 1.

### 2.3. Experimental Methods

#### 2.3.1. Physical Performance Tests and Storage Stability Test

Physical performance tests of asphalt include penetration, softening point, and ductility tests. Physical performance tests and storage stability tests of asphalt samples were, respectively, carried out according to Chinese standard test methods of asphalt and asphalt mixtures for highway engineering (T 0604-2011, T 0606-2011, T 0605-2011, and T 0661-2011).

#### 2.3.2. UV Aging Test

The prepared asphalt samples were placed on a Φ15 ± 0.5 mm stainless steel pan, which were subsequently transferred to a UV accelerated aging oven. This UV accelerated aging oven is (Model GM-UV-1140, Guangmai Instrument Equipment Co., Ltd., Dongguan, China) used UVA-340 fluorescent UV lamp as the UV radiation source. The radiation intensity and wavelength were 0.68 W/m^2^ and 340 nm, respectively. Their parameters were chosen according to the average equivalent sunlight intensity at noon in the local summer. The location is in Wuhan, Hubei Province, China.

**Figure 2:**
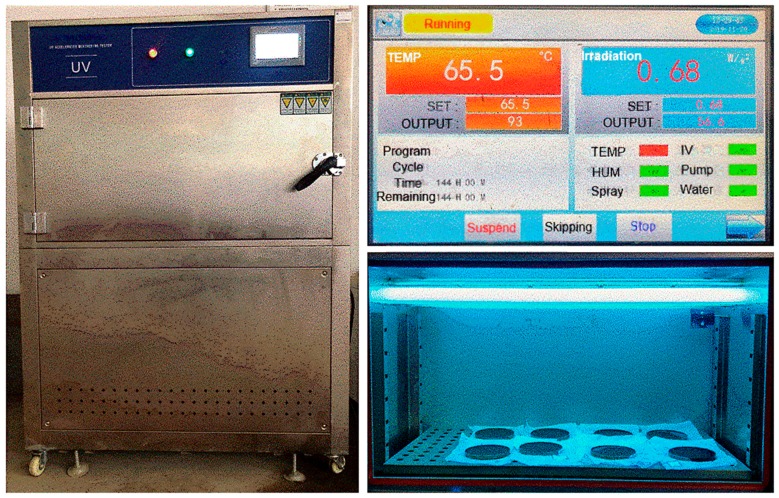
Photos of ultraviolet (UV) accelerated aging test.

Referring to “Plastics-Methods of Exposure to Laboratory Light Sources Part 3: Fluorescent UV Lamps” (ISO 4892-3: 1999) and (GB/T 16422.3-1997), 12 h of UV radiation at 65.5 ± 1 °C in the oven is equivalent to 30 days of outdoor UV radiation. Therefore, the UV aging time in the oven was set as 144 h, which is equivalent to 12 months of outdoor UV radiation. The humidity in the UV accelerated aging oven was 85% and the UV accelerated aging testing photos are shown in Figure 2.

#### 2.3.3. Gel Permeation Chromatography

GPC was used to determine the molecular size of asphalt according to different eluting times. During the test, the macromolecules were firstly eluted and then the micromolecules were eluted. The average molecular weight of asphalt samples was tested by GPC. The different molecular weight distribution ratios of the asphalt sample were calculated according to the molecular weight distribution curve of asphalt. The relationship between the molecular weight distribution and the physical properties of the asphalt sample was analyzed [28].

The asphalt samples were dissolved in tetrahydrofuran (THF) at a mass ratio of 1:400 and the solution concentration was 2.0 mg/mL. After the asphalt samples were completely dissolved in THF, they were filtered by 0.45 µm sieve and tested by PL-GPC50 gel chromatography with an HR 3 column and an HR 4E column at 35 °C [29].

#### 2.3.4. Fourier Transform Infrared Spectroscopy

Functional groups of different asphalt samples before and after UV aging can be determined by analyzing the position of the characteristic peak in infrared spectra. The aging index of a specific functional group can be calculated according to the corresponding peak area [30]. FTIR test was carried out by Nexus type Fourier transform infrared spectrometer (VEECO company, Somerset, NJ, USA). The infrared spectrum scanned from 4000 to 500^−1^ wavenumbers. The number of scans was 64 times and the resolution was 4 cm^−1^.

#### 2.3.5. Atomic Force Microscopy

Multimode PicoForce type AFM (Thermo Fisher, Waltham, MA, USA) is used to observe the topography of asphalt surfaces before and after UV aging [31]. Each asphalt sample was heated to a melted state and dropped on a 10 × 10 mm^2^ glass slide. Then, the glass slides were placed in an oven at 90 °C until asphalt samples flowed flat. Finally, glass slides were put into a dust-proof chamber and cooled to room temperature for the test.

## 3. Results and Discussion

### 3.1. The Physical Parameters Test Results of Asphalt before UV Aging

Based on the previous researches, the mass content of WEO or WPE in asphalts is usually within 10 wt % [32,33,34]. Therefore, the asphalt samples containing 2, 4, 6 and 8 wt % of WEO are abbreviated to 2%WEO, 4%WEO, 6%WEO, and 8%WEO. Meanwhile, the asphalt samples containing 2, 4, 6 and 8 wt % of WEO are abbreviated to 2%WPE, 4%WPE, 6%WPE, and 8%WPE. Three physical parameters (penetration, softening point, and ductility) of the matrix asphalt, WEO modified asphalts and WPE modified asphalts are shown in Table 2.

In the pink section of Table 2, it displays that the penetration and ductility of each WEO modified asphalt are larger than those of matrix asphalt, but the softening point of each WEO modified asphalt is lower than that of matrix asphalt. With the increasing contents of WEO, the penetration and ductility of WEO modified asphalt show an increasing trend while the softening point of WEO modified asphalt exhibits a decreasing trend. The results indicate that WEO softens the asphalts and enhances the low-temperature cracking resistance of asphalt.

In the yellow section of Table 2, it can be seen that the penetration and ductility of WPE modified asphalts are lower than those of matrix asphalt, while the softening point of WPE modified asphalt is higher than that of matrix asphalt. As the contents of WPE increase, the penetration and ductility of WPE modified asphalts decrease, while the softening point increases. Comparing with the matrix asphalt, WPE modified asphalt flows more difficult and becomes tougher. Therefore, high-temperature deformation resistance of WPE modified asphalt is improved.

As shown in the pink section of Table 3, the penetration and ductility of WEO/WPE mixture modified asphalts are smaller than those of matrix asphalt when the contents of WEO and WPE are the same. With the increase of the contents of the WEO/WPE mixture, the modified asphalts have smaller penetration and ductility but a more significant softening point. In addition, the ductility of WPE modified asphalt is lower than that of matrix asphalt, which is shown in Table 2. It indicates that the same content of WPE has a more considerable influence on asphalt’s ductility than WEO. The reason may be that WPE increases the discontinuous phase structure of the asphalt, thereby making the asphalt more easily broken.

In the yellow section of Table 3, 2% of WPE is mixed with different contents of WEO. As the content of WEO increases, the penetration and ductility of the WEO/WPE modified asphalt increase and the softening point decreases. This shows that the addition of WPE causes a significant reduction in asphalt ductility but WEO recovers the ductility of asphalt to some extent. In particular, 2%WPE + 4%WEO has similar penetration and softening point with those of matrix asphalt.

In the blue section of Table 3, the content of WEO in modified asphalt is 2% greater than that of WPE in modified asphalt. As the contents of WPE and WEO both increases, the penetration, and ductility of WEO/WPE modified asphalt decrease, but the softening point of WEO/WPE modified asphalt increases slightly. Based on the above results, it can be concluded that three similar physical parameters (penetration, softening point, and ductility) of the WEO/WPE modified asphalt are difficultly close to those of matrix asphalt. If only the penetration and softening point are considered, these two physical parameters of 4%WPE + 6%WEO and 6%WPE + 8%WEO are closest to those of the matrix asphalt.

### 3.2. GPC Analysis

Weight-average molecular weight, number-average molecular weight, and peak molecular weight of matrix asphalt, 6%WEO, and 6%WPE were determined by GPC. The molecular weight results of asphalt samples are shown in Table 4.

The weight-average molecular weight (*M*_w_) is the statistical average molecular weight by weight. The *M*_w_ of WPE modified asphalt is larger than that of WEO modified asphalt. The intermolecular forces become stronger due to the more substantial molecular weight of asphalt. Thus, the high-temperature deformation resistance is enhanced. The molecular weight polydispersity coefficient (d) of 6%WEO is the smallest among these three asphalts. This means the distribution range of different molecular weight is narrow and the molecular weight distribution is more concentrated. Therefore, the molecular phase changes within a specific range of molecular weight are more vigorous and the temperature sensitivity of asphalt is increased. The d value of 6%WPE is larger than that of 6%WEO. It indicates that the molecular weight becomes less concentrated. The peak molecular weight (*M*_p_) is the molecular weight of the most abundant component is asphalt. The *M*_p_ of 6%WEO is smaller than other asphalts. It implies that the content of low molecular weight components in WEO modified asphalt is relatively large, while the content of high molecular weight components is relatively small. The *M*_p_ of 6%WPE is larger than that of 6%WEO. It indicates that the content of low molecular weight components in WPE modified asphalt is relatively small, but the content of high molecular weight components in WPE modified asphalt is relatively large [35].

**Figure 3:**
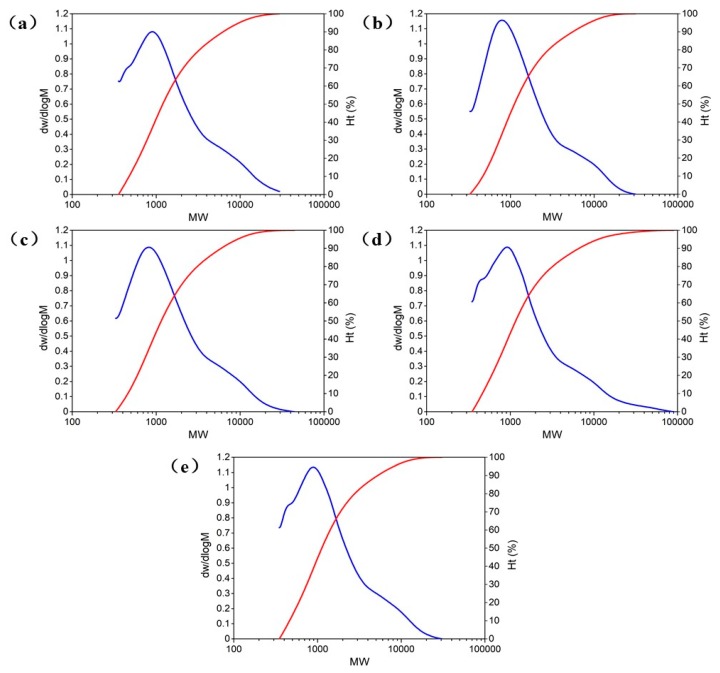
(**a**) Molecular weight distribution curves of matrix asphalt; (**b**) molecular weight distribution curves of 6%WEO; (**c**) molecular weight distribution curves of 6%WPE; (**d**) molecular weight distribution curves of 4%WPE + 6%WEO; (**e**) molecular weight distribution curves of 6%WPE + 8%WEO. The red curve is the integral distribution curve and the blue curve is the differential distribution curve.

The proportion of different molecular weight components in the total molecular weight of asphalt can be expressed by the distribution curve. The curve of weight fraction on the molecular weight is called the differential distribution curve and the curve of cumulative weight distribution on the molecular weight is called the integral distribution curve. Based on the physical properties test results of asphalt samples, 6%WEO, 6%WPE, 4%WPE + 6%WEO, and 6%WPE + 8%WEO with a similar performance to matrix asphalt were selected for GPC test. The molecular weight distribution curves of asphalt samples are shown in Figure 3. The red curve is the integral distribution curve and the blue curve is the differential distribution curve. The x-coordinate is the molecular weight (*M*_W_). The y-coordinate on the left is the weight fraction w (molecular content distribution) and the y-coordinate on the right is the cumulative weight distribution Ht.

According to the molecular weight distribution curves of asphalt samples, different molecular weight distribution ratios of asphalts can be calculated. The results are shown in Table 5.

According to the relevant researches on the molecular weight distribution of asphalt, the date in Table 5 is divided into three ranges: low molecular weight range (M < 4000), medium molecular weight range (4000–8000), and high molecular weight range (M > 8000) [36]. The results are shown in Table 6.

Table 6 shows that the molecular weight of the 6%WEO is more significant than that of the matrix asphalt in the low molecular weight range. The interaction forces between micromolecules are relatively small, which causes the changes in penetration, softening point and ductility in Table 2. The possible reasons are as follows. First, the smaller intermolecular force weakens the ability of asphalt to resist external damage, so that the depth of standard needle penetrated into asphalt increases. Second, the decrease in intermolecular force causes the asphalt molecules to undergo a relative displacement at a lower temperature so that the asphalt softens at a lower temperature. Third, the intermolecular cohesion of asphalt decreases, thus the plasticity and ductility of asphalt increase. Similarly, in the range of high molecular weight, the molecular weight percentage of 6%WPE is larger than that of 6%WEO. The interaction forces between more macromolecules become stronger, leading to changes in physical properties in Table 2.

In Table 6, it can also be seen that 4%WPE + 6%WEO has very similar proportions of low molecular weight component, medium molecular weight component and high molecular weight component with those of the matrix asphalt. This is consistent with the results of the physical properties test and also explains that 4%WPE + 6%WEO has a good potential to replace part of the matrix asphalt.

### 3.3. Storage Stability Test Results

The storage stability test results of WEO modified asphalt, WPE modified asphalt, WEO/WPE mixture modified asphalt are shown in Table 7.

As can be seen from Table 7, the storage stability of 4%WPE + 6%WEO is between those of 6%WEO and 6%WPE. Due to the similar components of WEO and the matrix asphalt, 6%WEO shows the best storage stability. However, 6%WPE is prone to segregation during heating storage, forming the PE-enriched phase and asphalt-enriched phase. Although high-speed shear stirring was applied to the WPE modified asphalt, the PE particles are easily attracted to each other and aggregated due to the polymeric structure and density. On the other hand, 4%WPE + 6%WEO has good compatibility with the matrix asphalt. The reason may be that WEO can enable the light components to promote the swelling of PE in the matrix asphalt [15]. Saturates, aromatics, and wax components in asphalt penetrate into the swelling PE molecules. Therefore, 4%WPE + 6%WEO forms a new colloidal structure with good storage stability.

### 3.4. Effect of UV Aging on Physical Properties of Asphalt

Physical parameters of asphalts before and after UV aging can be used to evaluate the aging degrees of asphalt. The penetration, softening point, and ductility of modified asphalt with different contents of WEO, WPE, WEO/WPE after the UV aging experiment are tested. The test results are shown in Table 8.

Comparing with the physical parameters of WEO modified asphalt before and after the UV aging experiment in the pink section of Table 8, it is found that physical parameters of WEO modified asphalt after the UV aging experiment have the same trend as those before the UV aging experiment. The WEO modified asphalt after the UV aging experiment has a lower penetration, softening point, and ductility than those before the UV aging experiment. However, the ductility of WEO modified asphalt after the UV aging experiment is still higher than that of the matrix asphalt after the UV aging experiment. The results indicate that the WEO modified asphalt hardens and the low-temperature crack resistance of WEO modified asphalt does not change after the UV aging experiment.

Comparing the physical parameters of WPE modified asphalt before and after the UV aging experiment in the yellow section of Table 8, it is seen that the physical properties of WPE modified asphalt after the UV aging experiment has the same change trend as those before the UV aging experiment. The penetration and ductility of WPE modified asphalt after the UV aging experiment are higher than that before the UV aging experiment, but the softening point decreases. Furthermore, the softening point of WPE modified asphalt after the UV aging experiment is still smaller than that of the matrix asphalt after the UV aging experiment. These results reveal that the WPE modified asphalt softens and the plasticity of WPE modified asphalt is enhanced after the UV aging experiment.

The physical parameters of 4%WPE + 6%WEO and 6%WPE + 8%WEO before and after the UV aging experiment are listed in the blue section of Table 8. It shows that the penetration of 4%WPE + 6%WEO after the UV aging experiment is less than that before the UV aging experiment, but the softening point and ductility of 4%WPE + 6%WEO after the UV aging experiment are higher than those before the UV aging experiment. In addition, the penetration and softening point of 6%WPE + 8%WEO after the UV aging experiment are higher than those before the UV aging experiment. While the ductility of 6%WPE + 8%WEO after the UV aging experiment is less than that before the UV aging experiment. To further reveal the influence of UV aging on the physical performance of asphalt, the aging indexes are useful to reflect physical parameters change during the UV aging process.

The aging indexes, including residual penetration ratio (RPR), softening point increment (SPI), and ductility retention ratio (DRR) of asphalt samples, are calculated according to the Equations (1), (2), and (3). The larger RPR and the DRR correspond to the smaller aging degree of asphalt. While the smaller SPI corresponds to the smaller aging degree of asphalt [37,38].

**Figure 4:**
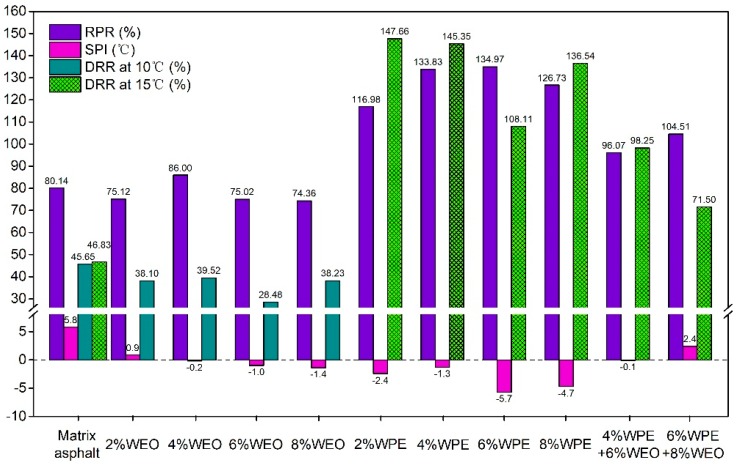
The aging indexes of asphalts.

Figure 4 shows that the RPR and DRR values of 2%WEO, 6%WEO, and 8%WEO are both lower than those of the matrix asphalt. However, the SPI values of all WEO modified asphalts are also lower than those of the matrix asphalt. Therefore, it is difficult to judge from the above results whether the WEO improves the UV aging resistance of asphalt. In particular, 4%WEO has a higher RPR value and smaller SPI value than those of the matrix asphalt.

(1) $$\mathrm{Residual}\;\mathrm{penetration}\;\mathrm{ratio}=\frac{\mathrm{Penetration}\;\mathrm{after}\;\mathrm{aging}}{\mathrm{Penetration}\;\mathrm{before}\;\mathrm{aging}}\times 100\%$$

(2) Softening point increment=Softening point after aging - Softening point before aging

(3) $$\mathrm{Ductility}\;\mathrm{retention}\;\mathrm{ratio}=\frac{\mathrm{Ductility}\;\mathrm{after}\;\mathrm{aging}}{\mathrm{Ductility}\;\mathrm{before}\;\mathrm{aging}}\times 100\%$$

The RPR and DRR values of WPE modified asphalt are greater than those of the matrix asphalt. Moreover, SPI values of WPE modified asphalt are smaller than those of the matrix asphalt and become negative after the addition of WPE. That means WPE can effectively improve the UV aging resistance of asphalt. It is worth noting that 6%WPE has a higher RPR value and a lower SPI value than those of the other WPE modified asphalts.

The RPR and DRR values of WEO/WPE modified asphalt are greater than those of the matrix asphalt, while the SPI value of WEO/WPE modified asphalt is smaller than that of the matrix asphalt. In addition, the DRR value of 4%WPE + 6%WEO is much higher than that of 6%WPE + 8%WEO and the SPI value of 4%WPE + 6%WEO is much lower than that of 6%WPE + 8%WEO. Therefore, it can be inferred that 4%WPE + 6%WEO has better UV aging resistance than the matrix asphalt and 6%WPE + 8%WEO.

### 3.5. FTIR Analysis

**Figure 5:**
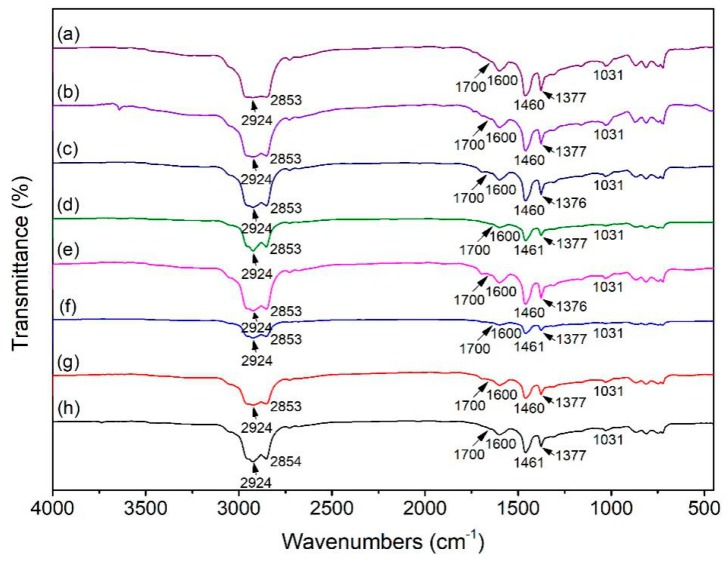
(**a**) Infrared spectrum of the matrix asphalt before the UV aging experiment; (**b**) infrared spectrum of the matrix asphalt after the UV aging experiment; (**c**) infrared spectrum of 4%WEO before the UV aging experiment; (**d**) infrared spectrum of 4%WEO after the UV aging experiment; (**e**) infrared spectrum of 6%WPE before the UV aging experiment; (**f**) infrared spectrum of 6%WPE after the UV aging experiment; (**g**) infrared spectrum of 4%WPE + 6%WEO before the UV aging experiment; (**h**) infrared spectrum of 4%WPE + 6%WEO after the UV aging experiment.

The infrared spectrum was used to analyze the changes of main functional groups’ absorption peaks of asphalt samples during the UV aging process. The infrared spectra of asphalt samples were shown in Figure 5.

The infrared spectra of asphalts are shown in Figure 5. The absorption peaks at 2924 cm^−1^ and 2853 cm^−1^ are the stretching vibration characteristic peaks of methylene –CH_2_– functional group. The absorption peak at 1600 cm^−1^ is the result of the stretching vibration of conjugated double bond C=C and carbonyl C=O on the benzene ring skeleton. The two firm absorption peaks at 1461 cm^−1^ and 1377 cm^−1^ are generated by stretching vibration of the asymmetric bond of C–CH_3_ and symmetric bond of –CH_2_–. The absorption peak at 1700 cm^−1^ is caused by the stretching vibration of carbonyl C=O, which is the characteristic peak of the functional group formed by asphalt aging. The strong absorption peak at 1031 cm^−1^ is caused by the vibration of the sulfoxide S=O functional group [39,40,41]. The infrared spectra peaks of WEO modified asphalt, WPE modified asphalt, and WEO/WPE mixture modified asphalt are basically the same as those of the matrix asphalt, indicating that WEO and WPE are physically blended with asphalt. The infrared spectra peaks of asphalts before and after the UV aging experiment are nearly the same, but the strengths are different.

The existing researches mainly used oxygen-containing functional groups (carbonyl and sulfoxide) as the aging characterization indicators of asphalt [42,43]. The carbonyl index (*I_C=O_*) and the sulfoxide index (*I_S=O_*) can reflect the changes of the oxygen-containing functional groups in the asphalt. The calculation formula of *I_C=O_* and *I_S=O_* of the asphalt are shown in Equations (4) and (5) [44]:

(4) $$I_{C=O}=\frac{A_{1700}}{\sum A}$$

(5) $$I_{S=O}=\frac{A_{1031}}{\sum A}$$

In Equations (4) and (5), A represents the peak area of the characteristic functional group. Σ*A* represents the peak area sum in a specific band range, and the value is the total spectral peak area sum of 4000–400 cm^−1^ range peaks. Σ*A* can be calculated by Equation (6).

(6) $$\sum A=A_{2924}+A_{2853}+A_{2728}+A_{1700}+A_{1600}+A_{1460}+A_{1377}+A_{1031}+A_{867}+A_{813}+A_{746}+A_{724}$$

The calculated results of the carbonyl index (*I_C=O_*) and sulfoxide index (*I_S=O_*) are shown in Table 9.

The saturates and aromatics are light components having a small molecular weight in asphalt, while the resins and asphaltenes are heavy components having a large molecular weight in asphalt. The UV aging of asphalt is the change process of the component in which intramolecular groups are irradiated by UV light with energy. As a result. the chemical bonds are broken and the asphaltenes component is formed by saturates, aromatics, and resins [45]. From Table 9, it can be seen that the carbonyl index and sulfoxide index of the matrix asphalt, WEO modified asphalt, and WPE modified asphalt are all increased after the UV aging experiment. That means the contents of carbonyl and sulfoxide in asphalt are increased. However, the contents of carbonyl and sulfoxide of 4%WPE + 6%WEO are decreased compared with that before the UV aging experiment. The reason may be that WEO provides the micromolecules in asphalts. On the other side, WPE absorbs some light components such as saturates and aromatics in asphalts. Thus, the swelling has occurred under the action of these light components and the micromolecules are produced due to the broken alkyl side-chain of WPE during the UV aging process [46]. The combination of WEO and WPE prevents the conversion of saturates and aromatics in asphalt during the UV aging process. Therefore, the formation of carbonyl and sulfoxide is reduced. It well explains that WEO/WPE improves on the UV aging resistance of asphalt from its chemical component change.

### 3.6. Atomic Force Microscopy Analysis

**Figure 6:**
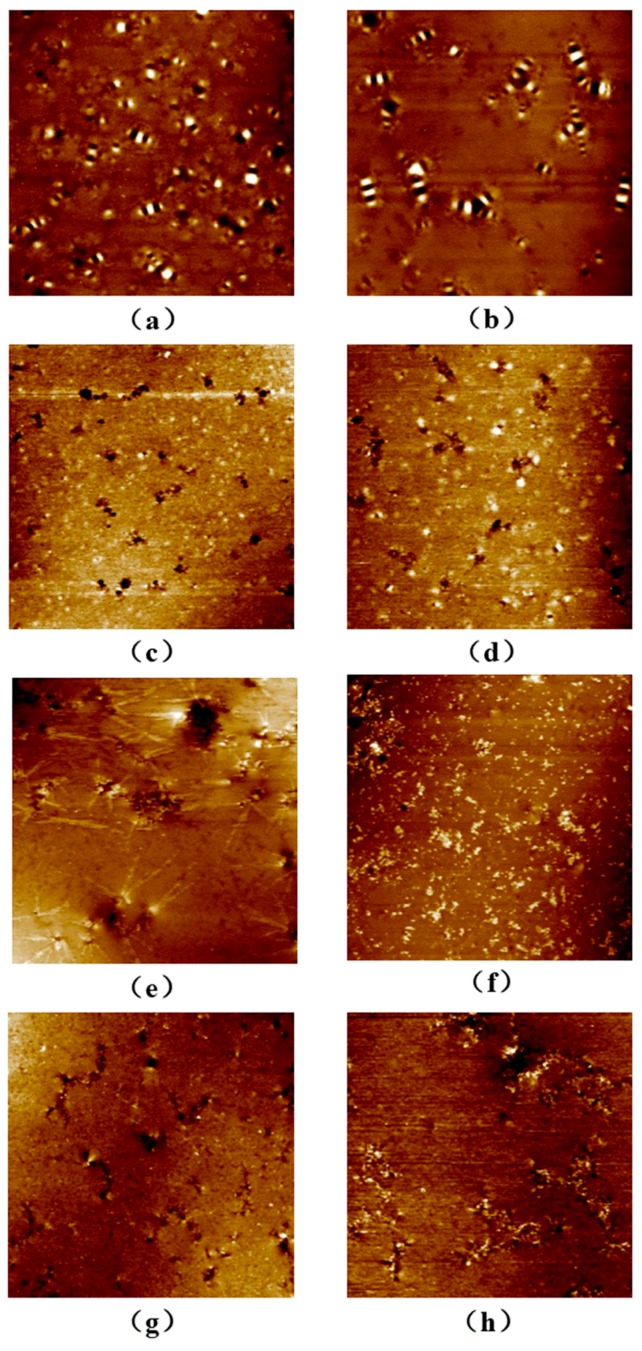
(**a**) The AFM morphology image of matrix asphalt before the UV aging; (**b**) the AFM morphology image of the matrix asphalt after the UV aging experiment; (**c**) the AFM morphology image of 4%WEO before the UV aging experiment; (**d**) the AFM morphology image of 4%WEO after the UV aging experiment; (**e**) the AFM morphology image of 6%WPE before the UV aging experiment; (**f**) the AFM morphology image of 6%WPE after the UV aging experiment; (**g**) the AFM morphology image of 4%WPE + 6%WEO before the UV aging experiment; (**h**) the AFM morphology image of 4%WPE + 6%WEO after the UV aging experiment.

AFM is widely used to observe the microtopography of the asphalt surface [47]. The morphologies of asphalts before and after the UV aging experiment are shown in Figure 6.

From Figure 6a,b, it can be seen that the “bee-like” structures of the matrix asphalt before and after the UV aging experiment appear in AFM morphologies. According to previous references, the “bee-like” structure of asphalt is formed by the association of strong polar asphaltene and high-molecular microcrystalline wax with some resins and a small number of oil components dispersed in the oil phase [48,49]. Changes in the components of the asphalt affect the size of the “bee-like” structure [50]. Compared with the asphalt before the UV aging experiment, the size of a single “bee-like” structure in the asphalt after the UV aging experiment becomes larger and the total number of “bee-like” structures decreases. As can be seen from Figure 6c,d, the fuzzy “bee-like” structures are scattered in the AFM morphology of WEO modified asphalt before the UV aging experiment. This is because the small molecules in the WEO fill the gaps between the matrix asphalt molecules, thereby diluting the asphaltenes and microcrystalline wax [51]. According to the molecular solution theory, this is equivalent to increasing the solvent content in the asphalt. After the UV aging experiment, there is a clear “bee-like” structure in the modified asphalt of WEO, but the size and number of “bee-like” structures are smaller than those of the aging matrix asphalt. Figure 6e,f shows that the WPE is dispersed in the matrix asphalt and the “bee-like” structure disappears. The reason may be that the aromatic hydrocarbon is a good solvent for the broken PE macromolecules. The aromatic hydrocarbon component in asphalt plays a swelling effect on PE. The incorporation of PE particles adsorbs light and saturated molecules and aromatic molecules in the asphalt. Swelling occurs under the action of the components so that the light components in the asphalt are relatively reduced [52]. In addition, PE can be dissolved in wax and the presence of wax in asphalt increases the swelling degree of PE. As a solvent, wax enters the PE molecular structure and changes the distribution of wax in asphalt. According to the molecular solution theory, it is equivalent to increasing the content of solute in asphalt. A small number of “bee-like” structures appear in the WPE modified asphalt after the UV aging experiment. Therefore, the size and number of the “bee-like” structure are smaller than the matrix asphalt after the UV aging experiment. From Figure 6g,h, it can be seen that the “bee-like” structure is not observed in the morphology of 4%WPE + 6%WEO before the UV aging experiment. This may be due to the combined effect of WEO and WPE on the matrix asphalt. Small molecules in WEO dilute asphaltenes and microcrystalline wax, while the WPE is adsorbed and swelled. In addition, the wax content in the asphalt can be used as a dispersant for PE. Some saturated, aromatic, and wax components in the asphalt enter the PE molecular structure. It increases in the relatively heavy components and limits the molecular flow in the asphalt. Based on the results of FTIR, the modification of the matrix asphalt by WEO and WPE is a physical blend process. WEO is a subsidiary product in the petroleum refining process. PE is a polymer compound formed by the polymerization of petroleum by-products. According to the “similar compatibility” principle, WEO/WPE can be stably dispersed in asphalt. After the UV aging process, no “bee-like” structure appears in the WEO/WPE modified asphalt. The reason may be that the UV aging of asphalt is a process of converting small molecules to large molecules. During the UV aging process, small molecules are provided by WEO and the chemical bonds of WPE are broken and degraded to produce small molecules. In this way, the polymerization of micromolecules in the asphalt can be slowed down to generate macromolecules, thereby improving the UV aging resistance of asphalt. Based on the above discussion, AFM morphologies of asphalts further reveal the microscopic mechanism of the WEO/WPE improving UV aging resistance of asphalt.

## 4. Conclusions

(1) Physical property results show that WEO can soften the matrix asphalt and enhance low-temperature crack resistance of asphalt. The WPE can make the matrix asphalt hard and increase the deformation resistance of asphalt at high temperatures. Physical properties of 4%WPE + 6%WEO and 6%WPE + 8%WEO are similar to those of the matrix asphalt. GPC experiments display that WEO increases the molecular weight of asphalt in the low molecular weight range, while WPE raises the molecular weight of asphalt in the high molecular weight range. The molecular weight distribution characteristic of 4%WPE + 6%WEO is most similar to that of the matrix asphalt and can effectively replace part of the matrix asphalt. The storage stability test indicates that 4%WPE + 6%WEO has satisfactory compatibility with the matrix asphalt.

(2) The physical aging indexes of asphalts reveal that 4%WEO or 6%WPE has better UV aging resistance than other WEO modified asphalts or WPE modified asphalts. Considering DRR and SPI, the UV aging resistance of 4%WPE + 6%WEO is superior to that of the matrix asphalt or 6%WPE + 8%WEO.

(3) The FTIR shows that WEO and WPE are physical blending modifications for asphalt. The contents of carboxyl and sulfoxide groups in matrix asphalt, WEO modified asphalt, and WPE modified asphalt after the UV aging experiment are increased, while the contents of carboxyl and sulfoxide groups in 4%WPE + 6%WEO after the UV aging experiment are decreased. The WEO and WPE in asphalt provide small molecules under the action of UV light, preventing the conversion of saturates and aromatics components in asphalt.

(4) The AFM morphologies exhibit that WEO and WPE can change the components of asphalt and reduce the formation of a “bee-like” structure. Finally, 4%WPE + 6%WEO can also reduce the formation of a “bee-like” structure after the UV aging experiment. The combined effect of WEO and WPE in asphalt can slow down the conversion of micromolecules to macromolecules during the UV aging process and thereby improve the UV aging resistance of asphalt.

## Figures and Tables

**Table 1:** The mass ratios of the matrix asphalt and different modifiers.

Asphalt Samples ^1^	Mass Ratios of Modifier to Asphalt (2 wt % )		
	Waste Engine Oil (WEO)	Waste Polyethylene (WPE)	Matrix Asphalt
2%WEO	2		98
4%WEO	4		96
6%WEO	6		94
8%WEO	8		92
2%WPE		2	98
4%WPE		4	96
6%WPE		6	94
8%WPE		8	92
2%WPE + 2%WEO	2	2	96
2%WPE + 4%WEO	4	2	94
2%WPE + 6%WEO	6	2	92
2%WPE + 8%WEO	8	2	90
4%WPE + 4%WEO	4	4	92
4%WPE + 6%WEO	6	4	90
6%WPE + 6%WEO	6	6	88
6%WPE + 8%WEO	8	6	86
8%WPE + 8%WEO	8	8	84

^1^ The abbreviations of differently prepared asphalt samples used in this paper are listed in the first column of this table.

**Table 2:** Physical properties of the matrix asphalt, waste engine oil (WEO) modified asphalts and waste polyethylene (WPE) modified asphalt before UV aging.

Asphalt Samples	Penetration (25 °C, 100 g, 5 s) (0.1 mm)	Softening Point (°C)	Ductility (5 cm/min, 10 °C) (cm)	Ductility (5 cm/min, 15 °C) (cm)
Matrix asphalt	86.1	49.6	23.0	99.5
2%WEO	101.3	45.0	29.4	
4%WEO	111.4	43.7	37.7	
6%WEO	138.9	41.8	64.6	
8%WEO	214.1	37.8	>100	
2%WPE	48.3	52.7		12.8
4%WPE	34.6	55.0		8.6
6%WPE	26.9	59.6		7.4
8%WPE	21.7	65.3		5.2

**Table 3:** Physical properties of the matrix asphalt and WEO/WPE mixtures modified asphalts.

Type of Asphalt	Penetration (25 °C, 100 g, 5 s) (0.1 mm)	Softening Point (°C)	Ductility (5 cm/min, 15°C) (cm)
Matrix asphalt	86.1	49.6	99.5
2%WPE + 2%WEO	64.6	51.7	16.3
4%WPE + 4%WEO	69.5	53.7	18.1
6%WPE + 6%WEO	66.9	54.3	6.9
8%WPE + 8%WEO	64.9	60.5	5.1
2%WPE + 4%WEO	87.9	47.9	23.7
2%WPE + 6%WEO	110.9	44.7	36.5
2%WPE + 8%WEO	131.0	44.6	58.4
4%WPE + 6%WEO	94.1	48.3	22.8
6%WPE + 8%WEO	84.3	49.5	20.0

**Table 4:** The molecular weights of the matrix asphalt, 6%WEO, and 6%WPE.

Type of Asphalt	Weight-Average Molecular Weight (M_w_)	Number-Average Molecular Weight (M_n_)	Coefficient of Dispersion (d)	Peak Molecular Weight (M_p_)
Matrix asphalt	2489	1005	2.4766	895
6%WEO	2228	957	2.3281	811
6%WPE	2395	963	2.4870	878

**Table 5:** Different molecular weight ratios (%) of asphalt samples.

Type of Asphalt	M ^a^ < 1000	1000–2000	2000–4000	4000–6000	6000–8000	8000–10,000	10,000–30,000	M > 30,000
Matrix asphalt	42.203	26.338	15.137	5.739	3.607	2.218	4.470	0
6%WEO	45.152	26.494	14.123	5.151	3.268	2.034	3.778	0
6%WPE	44.158	25.738	14.724	5.524	3.432	2.086	4.216	0.121
4%WPE + 6%WEO	43.523	26.217	13.953	5.156	3.238	1.995	4.833	1.085
6%WPE + 8%WEO	44.770	27.205	14.531	5.210	3.177	1.889	3.216	0.001

^a^ M represents the molecular weight.

**Table 6:** The molecular weight distribution of asphalt samples.

Type of Asphalt	Low Molecular Weight (%)	Medium Molecular Weight (%)	High Molecular Weight (%)
Matrix asphalt	83.678	9.346	6.958
6%WEO	85.769	8.419	5.812
6%WPE	84.620	8.956	6.423
4%WPE + 6%WEO	83.693	8.394	7.913
6%WPE + 8%WEO	86.506	8.387	5.106

**Table 7:** Storage stability test results of asphalt samples.

Type of Asphalt	Description
6%WEO	Crust and precipitation phenomena do not appear between asphalt.
4%WPE + 6%WEO	Slight crust appears on the rim of the penetration test container.
6%WPE	Crust appears on the entire surface between asphalt and penetration test containers. The thickness of the crust is more than 0.8 mm.

**Table 8:** Physical parameters of asphalts after the UV aging experiment.

Asphalt Samples	Penetration (25 °C, 100 g, 5 s) (0.1 mm)	Softening Point (°C)	Ductility (5 cm/min, 10°C) (cm)	Ductility (5 cm/min, 15 °C) (cm)
Matrix asphalt	69.0	55.4	10.5	46.6
2%WEO	76.1	45.9	11.2	
4%WEO	95.8	43.5	14.9	
6%WEO	104.2	40.8	18.4	
8%WEO	159.2	36.4	39.3	
2%WPE	56.5	50.3		18.9
4%WPE	46.7	53.7		12.5
6%WPE	36.0	53.9		8.0
8%WPE	27.5	60.6		7.1
4%WPE + 6%WEO	90.4	48.2		22.4
6%WPE + 8%WEO	88.1	51.9		14.3

**Table 9:** Characteristic function groups index of asphalt samples.

Type of Asphalt	$I_{C=O}$	$I_{S=O}$
Matrix asphalt	0.0036	0.0693
Matrix asphalt after the UV aging experiment	0.0045	0.0758
4%WEO	0.0018	0.0486
4%WEO after the UV aging experiment	0.0170	0.0621
6%WPE	0.0004	0.0410
6%WPE after the UV aging experiment	0.0195	0.0762
4%WPE + 6%WEO	0.0216	0.0708
4%WPE + 6%WEO after the UV aging experiment	0.0114	0.0702
